# An Indo-Pacific coral spawning database

**DOI:** 10.1038/s41597-020-00793-8

**Published:** 2021-01-29

**Authors:** Andrew H. Baird, James R. Guest, Alasdair J. Edwards, Andrew G. Bauman, Jessica Bouwmeester, Hanaka Mera, David Abrego, Mariana Alvarez-Noriega, Russel C. Babcock, Miguel B. Barbosa, Victor Bonito, John Burt, Patrick C. Cabaitan, Ching-Fong Chang, Suchana Chavanich, Chaolun A. Chen, Chieh-Jhen Chen, Wei-Jen Chen, Fung-Chen Chung, Sean R. Connolly, Vivian R. Cumbo, Maria Dornelas, Christopher Doropoulos, Gal Eyal, Lee Eyal-Shaham, Nur Fadli, Joana Figueiredo, Jean-François Flot, Sze-Hoon Gan, Elizabeth Gomez, Erin M. Graham, Mila Grinblat, Nataly Gutiérrez-Isaza, Saki Harii, Peter L. Harrison, Masayuki Hatta, Nina Ann Jin Ho, Gaetan Hoarau, Mia Hoogenboom, Emily J. Howells, Akira Iguchi, Naoko Isomura, Emmeline A. Jamodiong, Suppakarn Jandang, Jude Keyse, Seiya Kitanobo, Narinratana Kongjandtre, Chao-Yang Kuo, Charlon Ligson, Che-Hung Lin, Jeffrey Low, Yossi Loya, Elizaldy A. Maboloc, Joshua S. Madin, Takuma Mezaki, Choo Min, Masaya Morita, Aurelie Moya, Su-Hwei Neo, Matthew R. Nitschke, Satoshi Nojima, Yoko Nozawa, Srisakul Piromvaragorn, Sakanan Plathong, Eneour Puill-Stephan, Kate Quigley, Catalina Ramirez-Portilla, Gerard Ricardo, Kazuhiko Sakai, Eugenia Sampayo, Tom Shlesinger, Leony Sikim, Chris Simpson, Carrie A. Sims, Frederic Sinniger, Davies A. Spiji, Tracy Tabalanza, Chung-Hong Tan, Tullia I. Terraneo, Gergely Torda, James True, Karenne Tun, Kareen Vicentuan, Voranop Viyakarn, Zarinah Waheed, Selina Ward, Bette Willis, Rachael M. Woods, Erika S. Woolsey, Hiromi H. Yamamoto, Syafyudin Yusuf

**Affiliations:** 1 ARC Centre of Excellence for Coral Reef Studies, James Cook University, 1 James Cook Drive, Townsville, Queensland 4811 Australia; 2 School of Natural and Environmental Sciences, Newcastle University, Newcastle upon Tyne, NE1 7RU United Kingdom; 3 Experimental Marine Ecology Laboratory, Department of Biological Sciences, National University of Singapore, 16 Science Drive 4, 117558 Singapore, Singapore; 4 Smithsonian Conservation Biology Institute, Smithsonian Institution, Hawai’i Institute of Marine Biology, 46-007 Lilipuna Rd, Kaneohe, Hawaii 96744 USA; 5 National Marine Science Centre, Southern Cross University, 2 Bay Drive, Coffs Harbour, New South Wales 2450 Australia; 6 Oceans and Atmosphere, CSIRO, Queensland Biosciences Precinct, 306 Carmody Rd, St Lucia, Queensland 4072 Australia; 7 School of Biology, University of St Andrews, Sir Harold Mitchell Building, St Andrews, KY16 9TH United Kingdom; 8 Reef Explorer Fiji, Coral Coast Conservation Center, Votua Village, Korolevu, Nadroga Fiji; 9 Center for Genomics and Systems Biology, New York University Abu Dhabi, PO Box 129188, Abu Dhabi, UAE; 10 Marine Science Institute, College of Science, University of the Philippines, Velasquez Street, Diliman, Quezon City, Manila, 1101 Philippines; 11 Aquaculture, National Taiwan Ocean University, 2 Beining Rd, Keelung, 20224 Taiwan; 12 Reef Biology Research Group, Department of Marine Science, Faculty of Science, Chulalongkorn University, Phayathai Road, Bangkok, 10330 Thailand; 13 Biodiversity Research Center, Academia Sinica, 128 Academia Road, Section 2, Nankang, Taipei 11529 Taiwan; 14 Center of Excellence for the Oceans, National Taiwan Ocean University, 2 Beining Rd, Keelung, 20224 Taiwan; 15 Reef Guardian Sdn. Bhd., Bandar Tyng, Mile 6, North Road, Sandakan, Sabah 90000 Malaysia; 16 Smithsonian Tropical Research Institute, Apartado 0843-03092, Balboa, Republic of Panama; 17 Department of Biological Sciences, Macquarie University, Macquarie Park, New South Wales 2109 Australia; 18 Centre for Biological Diversity, University of St Andrews, St Andrews, KY16 9TH United Kingdom; 19 ARC Centre of Excellence for Coral Reef Studies, The University of Queensland, St Lucia, Queensland 4072 Australia; 20 The Mina & Everard Goodman Faculty of Life Sciences, Bar-Ilan University, Ramat Gan, 5290002 Israel; 21 Faculty of Marine Science and Fisheries, Syiah Kuala University, Banda Aceh, Aceh Indonesia; 22 Halmos College of Natural Sciences and Oceanography, Department of Marine and Environmental Science, Nova Southeastern University, 8000 N Ocean Drive, Dania Beach, Florida 33004 USA; 23 Evolutionary Biology and Ecology, Université libre de Bruxelles, Brussels, B-1050 Belgium; 24 Endangered Marine Species Research Unit, Borneo Marine Research Institute, Universiti Malaysia Sabah, Jalan UMS, Kota Kinabalu, Sabah 88400 Malaysia; 25 eResearch Centre, James Cook University, 1 James Cook Drive, Townsville, Queensland 4811 Australia; 26 Molecular & Cell biology, College of Public Health, Medical & Vet Sciences, James Cook University, 1 James Cook Drive, Townsville, Queensland 4811 Australia; 27 School of Biological Sciences, The University of Queensland, St Lucia, Queensland 4072 Australia; 28 Tropical Biosphere Research Center, University of the Ryukyus, 3422 Sesoko, Motobu, Okinawa, 905-0227 Japan; 29 Marine Ecology Research Centre, Southern Cross University, PO Box 157, Lismore, NSW 2480 Australia; 30 Department of Biology, Ochanomizu University, 2-1-1 Otsuka, Bunkyo-ku, Tokyo, 112-8610 Japan; 31 China-ASEAN College of Marine Sciences, Xiamen University Malaysia, Jalan Sunsuria, Bandar Sunsuria, Sepang Selangor, Darul Ehsan, 43900 Malaysia; 32 12 Rue Caumont, Saint-Pierre Reunion Island, 97410 France; 33 Centre for Sustainable Ecosystem Solutions and School of Earth, Atmospheric and Life Sciences, University of Wollongong, Northfields Avenue, Wollongong, New South Wales 2522 Australia; 34 Geological Survey of Japan, National Institute of Advanced Industrial Science and Technology, Tsukuba, Ibaraki 305-8567 Japan; 35 Department of Bioresources Engineering, National Institute of Technology, Okinawa College, 905 Henoko, Nago, Okinawa, 905-2192 Japan; 36 Graduate School of Engineering and Science, University of the Ryukyus, Nishihara, Okinawa 902-0213 Japan; 37 Glenala State High School, Durack, Queensland 4077 Australia; 38 Aquatic Science, Faculty of Science, Burapha University, 169 LongHaad Bangsaen Rd, Saensook, Mueang Chonburi 20131 Thailand; 39 Coastal and Marine Branch, National Biodiversity Centre, National Parks Board, 1 Cluny Road, Singapore, Singapore; 40 School of Zoology, Tel-Aviv University, Ramat Aviv, 6997801 Israel; 41 Department of Ocean Science, Hong Kong University of Science and Technology, Clear Water Bay, Kowloon, Hong Kong; 42 Hawai’i Institute of Marine Biology, University of Hawaii at Manoa, 46-007 Lilipuna Rd, Kaneohe, Hawaii 96744 USA; 43 Kuroshio Biological Research Foundation, 560 Nishidomari, Otsuki Town, Hata Kochi, 788-0333 Japan; 44 Reef Ecology Lab, Department of Biological Sciences, National University of Singapore, 16 Science Drive 4, 117558 Singapore, Singapore; 45 Department of Biological Sciences, National University of Singapore, 16 Science Drive 4, 117558 Singapore, Singapore; 46 School of Biological Sciences, Victoria University of Wellington, Wellington, 2820 New Zealand; 47 1952-3 Tomioka, Reihoku-machi, Kumamoto, 863–2507 Japan; 48 286/53-54 Suriwong Rd, Si Phraya, Bangrak, Bangkok, 10500 Thailand; 49 Department of Biology, Faculty of Science, Prince of Songkla University, 15 Karnjanavanich Rd, Hat Yai, 90110 Thailand; 50 Sustainable Research Vessel, Landéda, 29870 France; 51 Australian Institute of Marine Science, PMB 3, Townsville, Queensland 4810 Australia; 52 Institute for Global Ecology, Florida Institute of Technology, 150 West University Boulevard, Melbourne, Florida 32901-6988 USA; 53 25 Mettam Street, Trigg, Western Australia 6029 Australia; 54 Faculty of Science and Marine Environment, Universiti Malaysia Terengganu, Kuala Nerus, Terengganu 21030 Malaysia; 55 Red Sea Research Center, Division of Biological and Environmental Science and Engineering, King Abdullah University of Science and Technology, Thuwal, 23955-6900 Saudi Arabia; 56 Faculty of Agricultural Technology, King Mongkut’s Institute of Technology Ladkrabang, Chalongkrung Rd, Ladkrabang, Bangkok 10520 Thailand; 57 Tropical Marine Science Institute, National University of Singapore, 18 Kent Ridge Road, 119227 Singapore, Singapore; 58 College of Science and Engineering, James Cook University, 1 James Cook Drive, Townsville, Queensland 4811 Australia; 59 The Hydrous, PO Box 309, Sausalito, CA 94965 USA; 60 Okinawa Churashima Research Center, Okinawa Churashima Foundation, 888 Ishikawa, Motobu, Okinawa, 905-0206 Japan; 61 Faculty of Marine Science and Fisheries, Hasanuddin University, Makassar, Indonesia

**Keywords:** Marine biology, Conservation biology, Databases

## Abstract

The discovery of multi-species synchronous spawning of scleractinian corals on the Great Barrier Reef in the 1980s stimulated an extraordinary effort to document spawning times in other parts of the globe. Unfortunately, most of these data remain unpublished which limits our understanding of regional and global reproductive patterns. The Coral Spawning Database (CSD) collates much of these disparate data into a single place. The CSD includes 6178 observations (3085 of which were unpublished) of the time or day of spawning for over 300 scleractinian species in 61 genera from 101 sites in the Indo-Pacific. The goal of the CSD is to provide open access to coral spawning data to accelerate our understanding of coral reproductive biology and to provide a baseline against which to evaluate any future changes in reproductive phenology.

## Abstract (summary)

**Table:** 

Measurement(s)	Point in Time Date and Time Data Type • Spawning
Technology Type(s)	Observation • digital curation
Sample Characteristic - Organism	Scleractinia
Sample Characteristic - Environment	ocean
Sample Characteristic - Location	Pacific Ocean • Indian Ocean region

Machine-accessible metadata file describing the reported data: 10.6084/m9.figshare.13100552

## Background & Summary

Scleractinian corals are the ecosystem engineers of coral reefs, the most species-rich marine ecosystems. Scleractinian corals have a bipartite life history, with a sessile adult stage and a planktonic larval stage that allows dispersal among reefs. Corals produce larvae in one of two ways: gametes are broadcast-spawned for external fertilization or the eggs are retained for internal fertilization, followed by the release of planula larvae from the polyp. The discovery of multi-species synchronous spawning on the Great Barrier Reef^1^ stimulated a large effort to document coral spawning times in other regions of the world. Similar multi-species spawning events *sensu*^2^ have now been documented in over 25 locations throughout the Indo-Pacific^3–5^. However, much additional data on coral sexual reproductive patterns remain unpublished. Even when spawning data are published, there is often insufficient detail, such as the precise time and duration of spawning, to address many important questions. Consequently, predicting the month of spawning has been the focus of many studies to date^6^.

Coral spawning times can be used to address many significant and fundamental questions in coral reef ecology. Most coral species are notoriously difficult to identify and spawning times have been used to infer pre-zygotic barriers to fertilization and thus assist decisions about species boundaries^7,8^. While proximate cues associated with the month of spawning are reasonably well understood in some taxa^6,9^, the relationship between cues for the date and time of spawning are poorly understood. Similarly, potential phylogenetic patterns and geographical variation in spawning times are only beginning to be explored^10^. Knowing when corals spawn is also important for managing coastal development. For example, in Western Australia, legislation requires dredging operations to cease during mass spawning events^11,12^. Coral spawning is also an economic boon for tourist operators in many parts of the world, such as the Great Barrier Reef. Furthermore, population level records of spawning times provide a baseline against which to evaluate potential changes in spawning synchrony or seasonality associated with anthropogenic disruptions to environmental cues, in particular, sea surface temperature^13^. Knowledge of the timing of spawning is also essential for accurately estimating levels of connectivity among populations, given season differences in current flow^14^. The value of long-term species level data on coral spawning has recently been demonstrated in a test of the influence of temperature and wind on the night of coral spawning^15^.

**Fig. 1:**
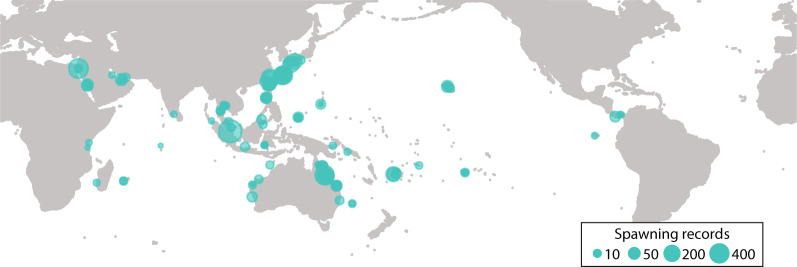
The number of spawning records by site.

In this data descriptor, we present the Coral Spawning Database (CSD). The CSD includes spawning observations for reef building coral species from the Indo-Pacific. The CSD includes 6178 observations (3085 of which were unpublished) of the time or day of spawning for 300+ scleractinian species in 61 genera (Online-only Table 1) from 101 sites (Fig. 1) in the Indo-Pacific. The goals of the CSD are: (i) to assemble the scattered and mostly unpublished observations of scleractinian coral spawning times and (ii) to make these data readily available to the research community. Our vision is to help advance many aspects of coral reef science and conservation at a time of unprecedented environmental and societal change.

## Methods

The CSD includes spawning times for broadcast spawning scleractinian coral species in the Indo-Pacific. There are two sources for these data: the literature and unpublished observations. Published literature was selected based on the authors’ knowledge of the subject area and a literature search using the terms “coral AND spawn*”. Over 50 researchers known by the authors to have extensive data on coral spawning times were approached to contribute unpublished data. This initial invitation led to a subsequent round of invitations to additional contributors. Of course, we encourage any researchers with data we have missed to contribute their observations in the annual update of the database. The database focusses on spawning times. Many other biological variables related to coral reproduction, such as fecundity, are available in the Coral Traits Database^16^.

**Fig. 2:**
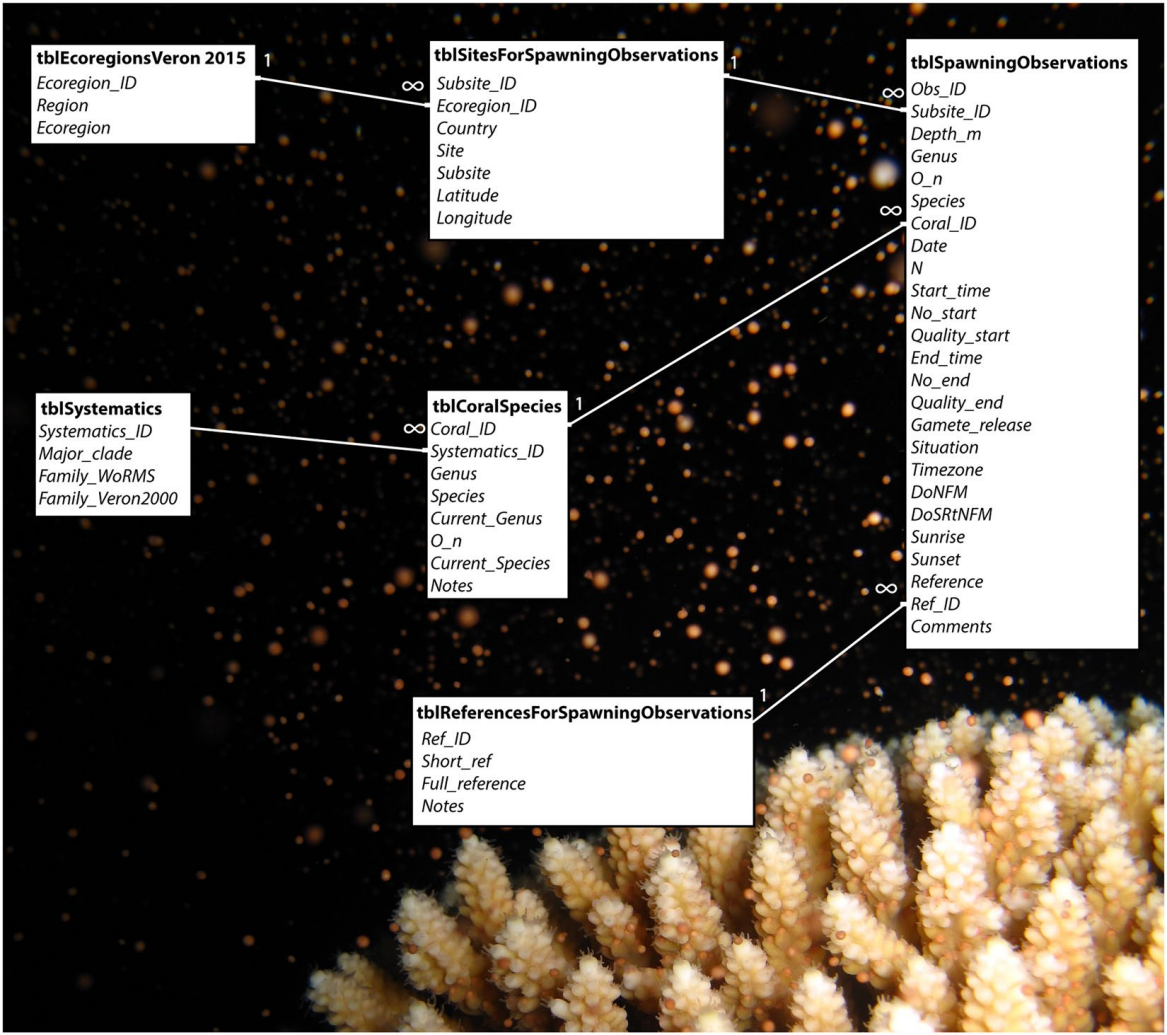
Arrangement of data tables in the Access relational database.

The database is available as a Microsoft Access relational database or an Excel spreadsheet. To minimise repetition in data entry, spawning observation information is entered in three primary tables (Fig. 2). The first (“tblSitesForSpawningObservations”) is used to enter geographic information on each study site; the second (“tblSpawningObervations”) contains details of the spawning activity recorded at each site; the third (“tblReferencesForSpawningObervations”) contains either full bibliographic details for published studies or details of the source of unpublished data. To assist with data analysis, three accessory tables are also linked. The first (“tblEcoregionsVeron2015”) allows sites to be grouped into the biogeographical Ecoregions proposed by^17^ or by broader region (e.g. Indian Ocean, Western and Central Pacific, Eastern Pacific). The remaining two tables allow the coral species to be grouped systematically for analysis. The first (“tblCoralSpecies”) has a list of over 1600 coral species with genus and species names (primarily from^18^ or subsequent descriptions of new species) mapped to currently accepted names (primarily from^19^) where the taxonomy has changed. The second (“tblSystematics”) allows species to be grouped into major clades or currently accepted families^19^ as revealed by molecular studies^20–22^.

### Data entry

#### Coral Spawning Database fields

1. **Site information (in tblSitesForSpawningObservations):**
  Ecoregion_ID link to Ecoregions (150) as defined by^17^
  Country            the country, territory (e.g. Guam) or island group (e.g. Hawaiian Islands) where spawning observation was made
  Site                      accepted name for broad geographical location (e.g. archipelago, island, offshore reef, bay, etc.) of the observation
  Subsite               more precise site name within location (where applicable; na entered where no subsite)
  Latitude             in decimal degrees (-ve values for sites South of the Equator).
  Longitude          in decimal degrees (-ve values for sites West of the Greenwich Meridian).
2. **Spawning observations (in tblSpawningObservations):**

Depth_m             the approximate depth at which the colony was collected (for *ex situ* observations) or observed (for *in situ* observations). If not recorded then −99 entered.

Genus                currently accepted genus name^19^

O_n                     open nomenclature qualifier: see explanation below under “Species identifications”.

Species               the species name used by the observer

Date                   date of spawning observation in the format day/month/year (e.g. 24/11/1983)

N                            number of colonies or individuals observed spawning. Used −99 if not known. If exact number of colonies not counted but more than a specific number were observed to spawn (e.g. > 25), then minimum number counted was entered (e.g. 25).

Start_time         time of first observation of spawning for colony(ies) of species: time (hh:mm) on a 24 hour clock e.g. 18:30. See “recording the time of spawning” below for ways to use the time fields to capture the various ways spawning is usually observed. No threshold applied to the intensity of spawning.

No_start                 no information on time that spawning started: True or False.

Quality_start   if No_start is False, Exact or Approx.

End_time          time of last observation of spawning for colony(ies) of species (if later than start time, normally): time (hh:mm) on a 24 h clock e.g. 18:30

No_end             no information on time that spawning ended: True or False

Quality_end    if No_end is False, Exact or Approx

Gamete_release (five character states as follows)

- Bundles – eggs and sperm released together packaged in bundles
- Eggs – only eggs released
- Sperm – only sperm released
- Both separately – eggs and sperm released separately from the same colony. Examples include *Lobophyllia hemprichii* and *Goniastrea favulus*
- Not recorded – release of gametes not observed or not reported

Situation            *In situ* = spawning observed underwater or *Ex situ* = spawning observed in tanks of colony(ies) recently removed from the reef.

Timezone         local time zone on the date of the spawning observation. This allows local time of spawning to be related to local time of sunset (or occasionally sunrise, for daytime spawners). This field is not an integer to accommodate 30 minute time differences (e.g. India and Sri Lanka are on UTC + 5.5). Enter -ve values for sites west of the Greenwich Meridian: e.g. −11 for Hawaii. (Note: Daylight Saving Times mean that time zones at some sites vary with date, e.g. Fiji goes from UTC + 12 to UTC + 13 from early November to early January).

The next four fields contain benchmarks for comparing spawning among sites for different species or groups of species^23^. The first is the date of the nearest full moon (DoNFM) to the date of spawning (with 75% of spawning recorded in the week after the full moon). This allows all spawning dates to be calculated in terms of days before or after the full moon (DoSRtNFM). Sunset provides a benchmark for comparing the times of spawning for most spawners (over 90% of spawning started within 4 hours of sunset) and sunrise for a few daytime spawners such as *Pocillopora verrucosa*. Dates of full moon and times of sunrise and sunset are available for given locations from the web (e.g. www.timeanddate.com) and can be entered manually. However, they can also be calculated automatically in the database based on the date, time zone and, for sunrise and sunset, the latitude and longitude. Excel spreadsheets are also available on request from the corresponding authors to calculate dates of full moon and times of sunrise and sunset in addition to a data entry template.

DoNFM            Date of Nearest Full Moon. Calculated automatically and corrected for longitude based on the local time zone.

DoSRtNFM     Date of Spawning Relative to Nearest Full Moon. Calculated automatically using time zone and date of observation in days before (-ve) or after ( + ve) the nearest full moon (ranges from −15 days to + 14 days).

Sunset                local time of sunset using a 24 h clock e.g. 18:30. Sunset and sunrise times were calculated for each observation based on latitude, longitude and time zone of the site and the date, using the method in the NOAA solar calculations day spreadsheet at https://www.esrl.noaa.gov/gmd/grad/solcalc/calcdetails.html. An Excel spreadsheet (Sunrise_Sunset_DoNFM_Calculations.xlsx) is provided for anyone wishing to use the Excel version of the dataset.

Sunrise              local time of sunrise using a 24 h clock e.g. 05:30. See above.

Ref_ID              a link to reference information for the data if available. If not the names of the observers are listed (e.g. Baird, Connolly, Dornelas and Madin unpublished)

Comments       any additional details provided

3) **Reference information (in tblReferencesForSpawningObservations):**

Each set of observations is referenced to its published or unpublished source in this table via a Ref_ID. The table contains two main fields: “Short_ref” (e.g. Baird *et al*. 2015) and “Full_reference” (e.g. Baird AH, Cumbo VR, Gudge S, Keith SA, Maynard JA, Tan C-H, Woolsey ES (2015) Coral reproduction on the world’s southernmost reef at Lord Howe Island, Australia. Aquatic Biology 23:275–284). These can be filled in before or after entering spawning observations. An email address is provided for all unpublished contributions.

### Notes to recording the time of spawning

For the quality of a start or end time to be ‘Exact’, a colony must be under continuous observation and the time of onset or end of spawning be observed and recorded. Most *in situ* observations would be expected to be approximate (‘Approx’).

The Quality_start, Quality_end, No_start and No_end fields are designed to accommodate the most common ways spawning is observed. A series of examples are given below.

1. A colony is observed spawning but it is not known exactly when it started. No end time is recorded.
  Here enter the time the colony was first observed spawning as the Start_time and the Quality_start as ‘Approx’. Leave the End_time blank and set No_end to True.
2. A colony is followed closely until spawning is observed to begin but the precise time when spawning ends is not recorded. However, the colony is observed to be still dribbling spawn 30 minutes after spawning started.
  Here enter the Quality_start_ as ‘Exact’ with the End_time set to 30 minutes after the Start_time and the Quality_end set to ‘Approx’.
3. A colony is followed closely from the beginning until the end of spawning.
  Here enter the times and note Quality_start and Quality_end as ‘Exact’.
4. A colony is placed in a bucket and checked every 30 minutes. At the first observation there is no evidence of spawning, 30 min later the surface of the water is covered in bundles and the colony is no longer spawning.
  Here enter the time of the first observation as the start time and the time of the second observation as the end time and set Quality_start and Quality_end to ‘Approx’.
5. Only the night of spawning is known, for example, gametes are no longer apparent in a tagged and sequentially sampled colony.

Here don’t enter either a start time or an end time and leave Quality_start and Quality_end blank. Set No_start and No_end to True.

### Species identifications

Species were generally identified following^18,24^ or by comparing skeletons to the type material or the original descriptions of nominal species. Specimens identified following^18,24^ were updated to the currently accepted names at the World Register of Marine Species^19^. The database also allows for uncertainties in species identifications to be indicated with the use of a series of open nomenclature qualifiers^25,26^ that allow the assignment of specimens to a nominal species with varying degrees of certainty. Specimens that closely resemble the type of a nominal species are given the qualifier cf. (e.g. *Acropora* cf. *nasuta*). Specimens that have morphological affinities to a nominal species but appear distinct are given the qualifier aff. (e.g. *Acropora* aff. *pulchra*): these specimens are either geographical variants of species with high morphological plasticity or potentially undescribed species. Species that could not be matched with the type material of any nominal species were labelled as sp. in addition to the location where they were collected (e.g. *Acropora* sp_1_Fiji). These specimens are most probably undescribed species. For 1% of records spawning colonies were only identified to genus (e.g. *Montipora* sp.). Contact the sources of these data for further information on the species identity.

## Data Records

A snapshot of the data contained in this descriptor can be downloaded from figshare^27^. The data includes 6178 observations, 3085 of which were unpublished with the remainder gleaned from the literature^28–128^. These data have been through a rigorous quality control and editorial process. Annual updates of the dataset will be uploaded to figshare as new version and also made available at any time on request from the Editor (JRG). Contributions to the CSD are welcome at any time and should be sent to the Editor (JRG).

## Technical Validation

The database is governed on a voluntary basis, by an Editor (JRG), Assistant Editors (JB & AGB), a Taxonomy Advisor (AHB) and a Database Administrator (AJE). Quality control of data and editorial procedures include:

1. **Contributor approval**. Database users must request permission to become a database contributor.
2. **Editorial approval**. Once a contributor sends data to the Editor, the data will be checked and if correctly formatted will be forward to the Database Administrator
3. **User feedback**. Data issues can be reported for any observation by email to the Editor

## Online-only Table

**Online-only Table 1:** Number of records by Genus.

Family	Genus	Species	records
Acroporidae	*Acropora*	119	2372
	*Alveopora*	1	2
	*Anacropora*	3	6
	*Astreopora*	4	25
	*Montipora*	38	480
Agariciidae	*Coeloseris*	1	3
	*Leptoseris*	1	8
	*Pavona*	8	73
Dendrophylliidae	*Duncanopsammia*	1	2
	*Tubastraea*	1	5
	*Turbinaria*	5	25
Euphylliidae	*Euphyllia*	2	26
	*Galaxea*	4	305
Incertae sedis	*Blastomussa*	1	1
	*Leptastrea*	2	16
	*Pachyseris*	2	27
	*Physogyra*	1	6
	*Plerogyra*	1	1
Poritidae	*Goniopora*	7	17
	*Porites*	12	207
Diploastraeidae	*Diploastrea*	1	18
Fungiidae	*Ctenactis*	2	60
	*Danafungia*	1	2
	*Fungia*	2	8
	*Heliofungia*	1	26
	*Herpolitha*	1	42
	*Lithophyllon*	4	39
	*Lobactis*	1	10
	*Pleuractis*	1	1
	*Polyphyllia*	1	1
	*Sandalolitha*	1	4
Lobophyllidae	*Acanthastrea*	4	36
	*Echinophyllia*	4	65
	*Homophyllia*	1	1
	*Lobophyllia*	8	64
	*Micromussa*	2	12
	*Moseleya*	1	2
	*Oxypora*	2	13
Merulinidae	*Astrea*	1	32
	*Australogyra*	1	4
	*Caulastraea*	2	10
	*Coelastrea*	2	93
	*Cyphastrea*	6	103
	*Dipsastraea*	12	218
	*Echinopora*	5	58
	*Favites*	14	532
	*Goniastrea*	8	197
	*Hydnophora*	4	29
	*Leptoria*	1	38
	*Merulina*	3	58
	*Mycedium*	3	36
	*Oulophyllia*	2	13
	*Paragoniastrea*	3	54
	*Pectinia*	4	71
	*Platygyra*	9	509
	*Scapophyllia*	1	16
Plesiastreidae	*Plesiastrea*	1	2
Pocilloporidae	*Palauastrea*	1	2
	*Pocillopora*	5	75
	*Stylophora*	1	4
Psammocoridae	*Psammocora*	2	13
	total		6178
